# Governance and policy in global neurosurgery: a scoping review of national and international efforts

**DOI:** 10.1007/s10143-025-03914-2

**Published:** 2025-10-31

**Authors:** Ellianne dos Santos Rubio, Khalil St. Brice, Felix Toussaint, Jimena Colado-Martinez, Garrett W. Thrash, Ali Azan Ahmed, James Kelbert, Walt Johnson

**Affiliations:** 1 Department of Neurosurgery, Curaçao Medical Center, Willemstad, Curaçao Curaçao; 2 University of the West Indies, St. Augustine, Trinidad and Tobago; 3 American University of the Caribbean School of Medicine, Cupecoy, Sint Maarten; 4 Epilepsy Clinic, Institute of Neurology and Neurosurgery, México City, México; 5 Department of Neurosurgery, College of Medicine, University of Arizona, Tucson, AZ USA; 6 Program of Global Surgery and Social Change, Harvard Medical School, Boston, MA USA; 7 Department of Neurosurgery, Loma Linda University, Loma Linda, CA USA; 8 Mercy Ships, Garden Valley, Lindale, TX USA; 9 University of Alabama at Birmingham Heersink School of Medicine, Birmingham, AL USA; 10 Medical College, Aga Khan University, Karachi, Pakistan; 11 Global Neurosurgery Research Group, Mission:Brain Foundation, Sausalito, CA USA

**Keywords:** Governmental organizations, Global neurosurgery, Global health, Health equity

## Abstract

Governmental organizations play a key role in making Global Neurosurgery timely, safe, and accessible. They are essential in decreasing disparity and improving outcomes through informing policy, funding, and resource allocation. The Boston Declaration aims to promote collaboration between high-income and low- and middle-income countries in addressing these challenges and realizing more equitable access to neurosurgical care worldwide. To identify and map key governmental stakeholders in Global Neurosurgery, emphasizing their roles in policy development, funding, resource allocation, research support, capacity building, advocacy, and international cooperation in addressing unmet neurosurgical needs. We employed a mixed-methods approach, analyzing the roles of governmental organizations in neurosurgery. A comprehensive literature review was conducted using PubMed, Embase, and Global Index Medicus. Sources included Ministries of Health and National Institute of Neurological Disorders and Stroke (NINDS) websites, annual reports, social media, and world health organisation (WHO) documents. Inclusion criteria covered governmental organizations and health policy institutions; non-governmental organizations and those without documented impact were excluded. WHO was the most frequently cited organization (25%) guiding global neurosurgical policy. National Ministries of Health (23%) were pivotal in developing policy based on country-specific needs. Centers for Medicare and Medicaid Services were cited in 18%, reflecting their funding role in the U.S., while military health systems (7%) were accountable for trauma care. International partnerships, though less frequent (5%), played vital roles in improving access in low-resource settings. Government agencies like the WHO, Ministries of Health, and Centers for Medicare and Medicaid Services (CMS) advance Global Neurosurgery via policy, financing, and service provision. This underscores the urgent need for sustained collaboration, implementation teams, and accountability to achieve long-term equity.

**Supplementary information:**

The online version contains supplementary material available at 10.1007/s10143-025-03914-2.

## Introduction

 The Bogotá Declaration marked a watershed moment in the global advocacy for neurosurgical services with the launch of the Global Action Plan in 2016 [1]. This initiative fundamentally redefined neurosurgery, not only as a specialized medical field, but as an essential component of global health. This is further evidenced by the increasing literature devoted to the challenges centered around neurosurgical care [2–5], the establishment of Global Neurosurgery-oriented organizations [6], and the addition of Global Neurosurgery sessions to various international meetings [7]. This momentum culminated in the Boston Declaration (April 24, 2025), where over one hundred governmental and non-governmental entities collectively affirmed neurosurgery as a global public good, calling for coordinated investment, workforce expansion, and policy integration. Actionable commitments such as an increase in educational efforts to enhance the neurosurgical workforce, more funding for resources, publication and research opportunities etc.

This manuscript maps the evolution of government agencies and their influence on the governance of neurosurgical services and organizations. To achieve this, we identify the key roles in these agencies, review agency policy, and examine the resources devoted to the programs. These factors will be examined in terms of their relevance to the evolution of Global Neurosurgery. In this way, the paper attempts to demonstrate how policymaking in these governments has been an important factor in the expansion of neurosurgery as an essential health sector beyond the confines of individual countries.

## Materials & methods

A mixed-methods approach was employed to examine the role of governmental organizations in shaping Global Neurosurgery policies and governance frameworks over time. This approach provides a comprehensive understanding of both qualitative insights and quantitative data relevant to the field of Global Neurosurgery.

### Search strategy and sources

A systematic literature search was conducted across three primary databases: PubMed, Embase, and Global Index Medicus, *from database inception (PubMed 1872) to October 2024*, following *the PRISMA-ScR (Preferred Reporting Items for Scoping Reviews) guidelines and the Joanna Briggs Institute (JBI) methodology framework.*. The search strategy was designed to capture a wide range of studies related to both neurosurgery and governance. For example, in PubMed, the search terms included “neurosurgery” and “governance,” combined with “policy,” “governmental agencies,” and related MeSH terms. Similar search strategies were applied in the other databases to ensure comprehensive coverage. The complete PubMed search string and database-specific adaptations are provided in Supplementary Table 1.

To supplement the peer-reviewed literature, we also searched the WHO’s IRIS database to gather policy documents and organizational reports relevant to global health governance, explicitly focusing on neurosurgery. In total, 283 articles were identified across the four primary databases.

### Grey literature search

Grey literature records retrieved from Global Index Medicus and institutional websites were screened in parallel with peer-reviewed sources and are included in the PRISMA flow diagram totals. This search used official websites, reports, and bulletins from global health ministries, international organizations, and governmental health agencies. We employed targeted search terms such as “Ministry of Health,” “neurosurgery,” “government,” and the names of specific countries to identify relevant documents and ongoing governmental initiatives. This allowed us to incorporate up-to-date information on the role of governments in developing Global Neurosurgery.

### Study selection and inclusion criteria

Studies and documents were selected based on predefined inclusion and exclusion criteria. The inclusion criteria encompassed governmental organizations at local, national, continental, and global levels, institutions that fund neurosurgical capacity-building initiatives, and public health organizations contributing to policy development related to Global Neurosurgery. Additionally, we included reports and communications from international collaborations that directly impacted neurosurgical services in low- and middle-income countries (LMICs).

We excluded non-governmental organizations (NGOs), private sector entities, and academic institutions unless they were directly involved in governmental policymaking or had documented participation in public health initiatives related to neurosurgery. A complete list of the stakeholders and organizations included is provided in the supplementary materials.

### Data extraction and analysis

Once relevant studies and grey literature were selected, we used Rayyan AI [8] to facilitate the blind screening and data extraction. Authors F.T. and K.S. independently conducted the initial screening. Any discrepancies between the two reviewers were resolved through discussion, reaching a consensus on the final selection of documents.

For each relevant document, we extracted key data related to the role of governmental organizations in developing and implementing neurosurgery policies. This data included information on policy frameworks, partnerships, and specific programs to address gaps in neurosurgical services in LMICs.

**Fig. 1:**
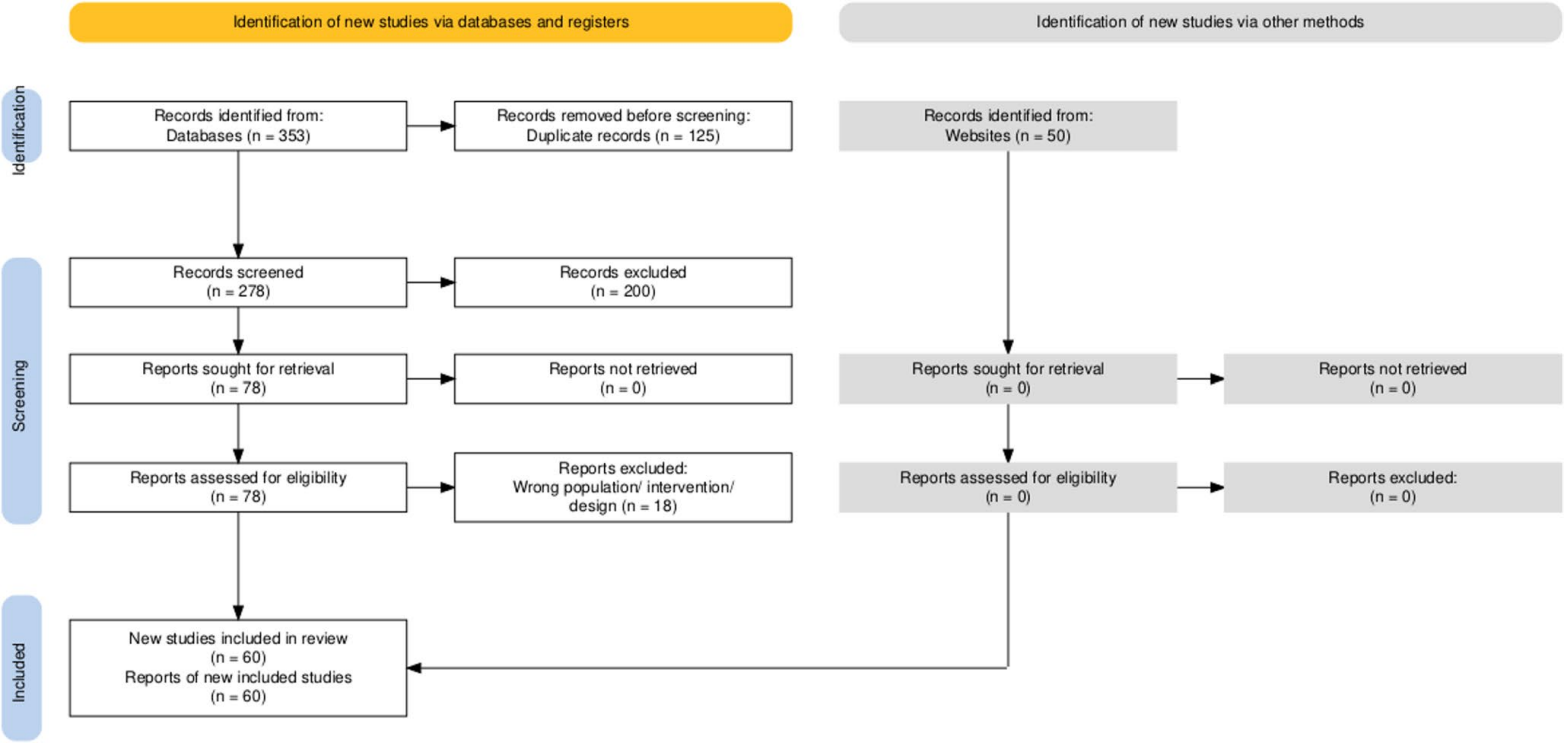
PRISMA diagram illustrating article screening and exclusion criteria

The extracted data was systematically entered into a spreadsheet for further analysis. We categorized the organizations based on their role in Global Neurosurgery, level of influence in policymaking, and the extent of their financial investment in neurosurgical programs. This categorization allowed us to identify trends in governmental involvement across different regions and assess the impact of various initiatives on global neurosurgical development (Fig. 1).

## Synthesis of results

The synthesized data was analyzed qualitatively to identify recurring themes related to governmental involvement in Global Neurosurgery. Key areas of focus included:

1. Policy development: How governments have integrated neurosurgery into national health policies.
2. Collaborations: The role of intergovernmental and international partnerships in advancing neurosurgical services in LMICs.
3. Impact: What has been the impact of governmental involvement on Global Neurosurgery.

Additionally, we used a framework to assess the evolution of Global Neurosurgery governance over time, evaluating shifts in policy priorities and the effectiveness of funding mechanisms. The results were presented in a structured table, categorizing the organizations by their roles and contributions to the field.

## Results

**Fig. 2:**
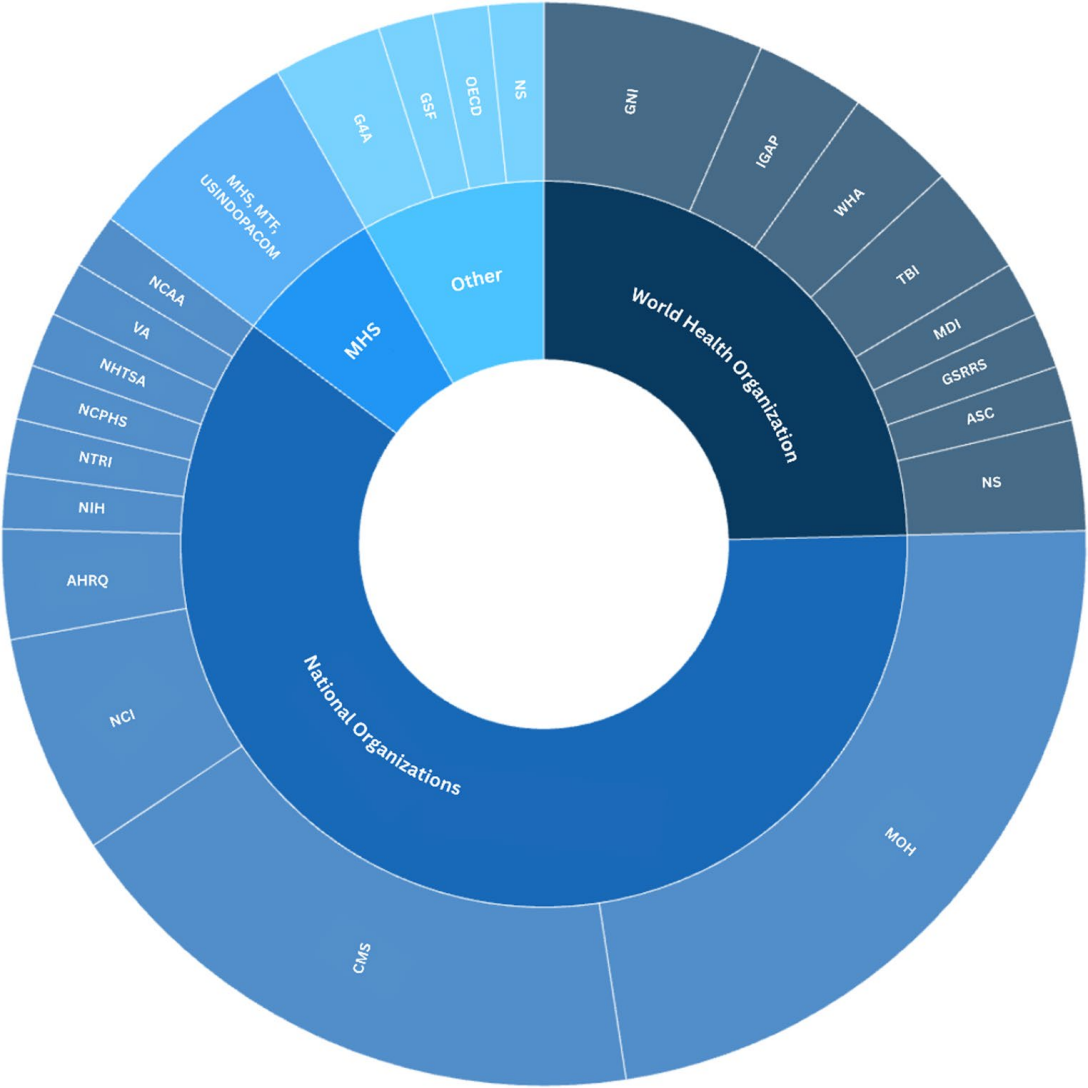
Stakeholder mapping of world and national health organizations. This is a qualitative visual representation of stakeholder frequency, with sector sizes reflecting proportional citation frequency. GNI = Global Neurosurgery Initiative, IGAP = International Global Action Plan on Epilepsy, WHA = World Health Assembly, TBI = Collaborating Centre Task Force on Mild Traumatic Brain Injury, MDI = Minimum Dataset for Injury, GSRRS = Global Status Report on Road Safety, ASC = African Subcommittee, WHO = World Health Organization, MOH = Ministries of health, CMS = Centers for Medicare & Medicaid Services, NCI = National Cancer Institute, AHRQ = Agency for Healthcare Research and Quality, NIH = National Institutes of Health, NTRI = National Trauma Research Institute, NCPHS= National Commission for the Protection of Human Subjects, NHTSA = National Highway Traffic Safety Administration, VA = Department of Veteran Affairs, NCAA = National Collegiate Athletic Association, MHS = Military Health Systems, MHT = Military Treatment Facilities, USINDOPACOM = US Indo-Pacific Command, G4A = G4 Alliance, GSF = Global Surgery Foundation, OECD = Organization for Economic Cooperation and Development, NS = Not Specified

Here, we summarize the 60 articles after PRISMA screening and investigate the major stakeholders of governmental organizations in Global Neurosurgery (Fig. 2). Across the 60 included articles, WHO was referenced in 25%, national Ministries of Health in 23%, CMS in 18%, military health services in 7%, and international coalitions such as G4 Alliance and *International Global Action Plan on Epilepsy* (IGAP) in 5%. A detailed breakdown of all included studies, including study type, region, organization type, and scope of contribution, is provided in Table 1.

**Table 1:** Summary of included studies

Author	Region	Organisation Type	Contribution
Asija et al. 2024	South Asia (India, Nepal - LMIC survey)	**Academic or Hospital-Led Policy Effort** (Neurosurgical Departments/Respondents)	**Functional Neurosurgery** - Training Gap/Affordability/Access Barriers
Zaidi et al. 2024	South Asia (Pakistan)	**National Ministries & Public Health Agencies** (MOH Pakistan + Academic Partner)	**Stroke Systems** - Lack of Units/Delayed Referral/No National Strategy
Shakir et al. 2024	Global South (LMICs)	**National Ministries & Public Health Agencies** (MOH/National Insurance Bodies)	**Brain Tumor Care** - Underfunding/Policy Instability/No Reimbursement
Tini et al. 2024	Global South (LMICs; examples include Ukraine, Philippines)	**National Ministries & Public Health Agencies** (governments; UHC packages); **Multilateral Coalitions/Advocacy Platforms** (WHO initiatives referenced in citations)	**Neuro-oncology (GBM)** - Policy levers across NSOAP domains: insurance/UHC inclusion, regulate RT/drugs; build registries, labs, workforce; enable trials/telemedicine
Gupta et al. 2024	Global (LMIC emphasis)	**Global Health Agencies** (WHO - IGAP/WHA)	**Epilepsy/neurology policy** - positions neurosurgery within WHO’s IGAP; outlines monitoring/implementation needs and opportunities for neurosurgical engagement.
Kim et al. 2023	Southeast Asia (Cambodia - Phnom Penh)	**National Ministries & Public Health Agencies** (major government hospital; residency program)	**Neurosurgical Burden** - Trauma-dominant caseload; workforce shortage; capacity building
Woodle et al. 2023	Asia-Pacific (U.S. Military facility in Okinawa, Japan)	Military Health Systems (U.S. Department of Defense/Military Treatment Facilities)	**Pediatric Neurosurgery Delivery -** Demonstrates feasibility of high-level neurosurgical care within overseas military hospitals; supports military readiness policy
Pattisapu et al. 2023	Global South (LMIC focus - Africa & Asia)	**Multilateral Coalitions/Advocacy Platforms** (G4 Alliance, WFNS, Global Surgery Networks)	**Neurosurgical Advocacy** - Global policy voice; NSOAP inclusion; representation in WHO processes
Salisu-Kabara et al. 2022*	West Africa (Nigeria)	**Academic or Hospital-Led Policy Effort** (Tertiary Hospital Surgical Unit)	**Traumatic Brain Injury (TBI)** - Limited neurosurgical workforce; Delayed referrals; Lack of critical care infrastructure
Aruah et al. 2023	West Africa (Nigeria)	**National Ministries & Public Health Agencies** (Federal Ministry of Health Nigeria)	**Spinal Trauma** - Inadequate rehabilitation services; Poor policy implementation; Limited funding for post-surgical care
Javed et al. 2023	South Asia (Pakistan)	**Global Health Agencies** (WHO/WHA; PGSSC); **National Ministries & Public Health Agencies** (MOH Pakistan; NVSC2025)	**System strengthening (neurotrauma)** - Pakistan WHA68.15 signatory; NVSC2025 developed with WHO/PGSSC; calls for equitable public-sector neurosurgical access and policy follow-through.
Tebha et al. 2023	LMICs (Africa, Asia, Americas)	National Ministries & Public Health Agencies (MOH-Oriented Review Framework)	**Glioblastoma** - Radiotherapy & Workforce Shortage/High Cost Burden
Garcia et al. 2023	Global (WHA75)	**Global Health Agencies** (WHO/WHA; IGAP); **Multilateral Coalitions/Advocacy Platforms** (G4 Alliance; GSF; GAPSBi-F)	**Global advocacy** - neurosurgical delegation at WHA75; push for folic-acid fortification resolution; highlight IGAP (epilepsy/neurologic disorders) and financing via GSF.
Shlobin et al. 2023	Multi-Regional (Latin America, Africa, Asia)	**Global Health Agencies** (WHO/GAPSBiF)	**Spina Bifida Prevention** - Folic acid fortification advocacy; neurosurgeons in prevention policy
Barthélemy et al. 2022	Global South (LMICs)	**National Ministries & Public Health Agencies** (LMIC health ministries; WHO benchmarking)	**Neurotrauma Surveillance** - National registry data dictionaries; WHO alignment; infrastructure gaps
Veerappan et al. 2022	Global (Case Studies Across LMICs)	**Multilateral Coalitions/Advocacy Platforms** (UN SDG3, Harvard PGSSC, WFNS)	**Public Health Integration** - Food fortification; RTA legislation; neurosurgeon-led advocacy models
Jamjoom et al. 2022	Middle East (Saudi Arabia)	**National Ministries & Public Health Agencies** (MOH + Military & Private Hospitals)	**Workforce Distribution** - Regional neurosurgeon disparity; recommends redistribution strategies
Goyal et al. 2022	North America (USA)	**National Registry/Regulatory Bodies** (CoC/NCDB)	**Glioblastoma Surgery** - Access disparities at high-volume CoC centers; race/socioeconomic inequity
Masiliūnas et al. 2022	Europe (Lithuania)	**National Ministries & Public Health Agencies** (MOH/national stroke program)	**Acute Ischemic Stroke** - Comprehensive national policy; expanded reperfusion access; door-to-needle improvement
Rolle et al. 2022	Caribbean (CARICOM member states)	**National Ministries & Public Health Agencies** (helmet legislation; enforcement systems)	**Traumatic Brain Injury** - Helmet safety governance; laws & enforcement; infrastructure/regulations
Singer et al. 2022	Global	**Global Health Agencies** (WHA/WHO); **Multilateral Coalitions/Advocacy Platforms** (GAPSBi-F; FFI; IFSBH)	**NTD prevention (folic acid fortification)** - urges WHA resolution; 58 countries mandate fortification; advocates pediatric-led policy action and monitoring.
Jella et al. 2021	North America (USA)	**Health Financing & Reimbursement Bodies** (CMS/MACRA)	**Low-Back Pain Imaging** - OP-8 metric; MACRA-linked reimbursement; utilization trends by hospital type
Lepard et al. 2021	Global (HIC vs. LMIC)	**National Ministries & Public Health Agencies** (helmet legislation)	**Road-Traffic Injury Prevention** - Mandatory helmet laws; improved outcomes; policy effectiveness by income level
Morii et al. 2021	East Asia (Japan - Hokkaido)	**Academic or Hospital-Led Policy Effort** (endovascular stroke network evaluation)	**Acute Ischemic Stroke** - Drive-and-Retrieve thrombectomy model; access expansion; cost-effectiveness
Lefever et al. 2021	North America (USA)	**Professional Society/Coalition** (AANS/CNS Washington Office; advocacy to government)	**US Neurosurgery Advocacy** - Legislative process; lobbying/PACs; federal & state policy engagement
Tate et al. 2021	Global (multisite; militaryrelevant)	**Military & Defense Health Systems** (DoD/VA collaboration; ENIGMA Military WG)	**Traumatic Brain Injury** - Multi-site harmonization; data sharing; research coordination
Kim et al. 2020	North America (USA)	National Ministries & Public Health Agencies (NCI/NIH)	**Brain Metastasis** - Federal Research Strategy/Coordination Gaps
Antony et al. 2020	Oceania (Australia)	**National Ministries & Public Health Agencies** (State/Territory Health Departments; public hospitals)	**COVID-19 service delivery** - Elective case reductions; urgent care maintained; triage & preparedness lessons
Richter et al. 2020	North America (USA)	Health Financing & Reimbursemen t Bodies (CMS/Medicare; ACOs; bundledpayment programs)	**Reimbursement policy** - Inflation-adjusted neurosurgery payments declined; bundled-payment context; service viability
Dallas et al. 2019	North America (USA)	**National Ministries & Public Health Agencies** (AHRQ/HCUP–NIS; payer mix incl. Medicare/Medicaid/private)	**Pediatric NMS fusion** - High median cost; regional/hospital variation; payer differences
McClelland III et al. 2019	North America (USA)	**Health Financing & Reimbursement Bodies** (CMS/Medicare; ATRA policy)	**SRS payment policy** -ATRA altered Gamma Knife vs. LINAC parity; utilization shifts
Childers et al. 2019	North America (USA)	Health Financing & Reimbursemen t Bodies (CMS/RVU system); Academic or Hospital-Led Policy Efforts (ACS-NSQIP registry)	**Payment policy (RVUs)** - shows variation in work RVUs explained by objective measures (OR time, LOS, readmission/reoperation); recommends using registry data to improve RVU updates and reduce specialty differences.
Rumalla et al. 2018 *	North America (USA)	**Health Financing & Reimbursement Bodies** (CMS/Medicare; insurance schemes)	**Epilepsy readmissions** - national database analysis of drivers of 30-day readmission; payer/insurance and clinical factors inform reimbursement and quality policy.
Khan et al. 2019	South America & South Asia (Chile; Pakistan)	**National Ministries & Public Health Agencies** (National disaster/health ministries; WHO Emergency Medical Teams standards; UN/NGO partners)	Disaster/trauma systems - Mass Casualty Centres model; civilian–military integration; 24/7 surgical capacity
Yousefzadeh-Chabok et al. 2018	Middle East (Iran)	**National Ministries & Public Health Agencies** (MOHME; national trauma registries)	**Cerebrovascular/trauma systems** - national registry initiatives (INTRD; NSCIR) and hospital data processes; highlights standardized data capture for policy/surveillance.
Cancedda et al. 2018	Sub-Saharan Africa (Rwanda)	**National Ministries & Public Health Agencies** (Rwanda MOH; CDC/PEPFAR); **Multilateral Coalitions/Advocacy/Donors** (Global Fund); **Academic or Hospital-Led Policy Efforts** (U.S. universities/teaching hospitals)	**Health workforce & systems** - government-led HRH scale-up with external partners; documents policy/financing model and outcomes for training, retention, and service delivery.
Foster et al. 2018	Global (HIC emphasis)	**Academic or Hospital-Led Policy Efforts (without formal mandate)** (universities/clinical guideline groups)	**Low back pain policy**; align practice to evidence; prioritize self-management/exercise; curb routine imaging/opioids/surgery; redesign pathways & payment/public-health strategies.
Yoon et al. 2018	North America (USA)	**Health Financing & Reimbursement Bodies** (Insurers; state price tools)	**Price transparency/value** - large cost variation in neurosurgery; promote “expected payment” episode data; pair with scorecards, reference pricing.
Ostrom et al. 2017	North America (USA)	**National Ministries & Public Health Agencies** (NCI/NIH); **Academic or Hospital-Led Policy Efforts** (institutional biobanks/consortia)	**Neuro-oncology biobanking** - governance, consent, linkage to registries; recommends standardized policies and national coordination to advance glioma/GBM research.
Villelli et al. 2017	North America (USA-Massachusetts vs. New York control)	**Health Financing & Reimbursement Bodies** (Medicare/Medicaid/private insurers)	**Spine surgery & reform**; MA reform shifted payer mix-fewer uninsured/private, more Medicare; treated cohort older.
Menger et al. 2017	North America (USA)	**Health Financing & Reimbursement Bodies** (private insurers; Medicare/Medicaid; TRICARE; workers’ comp)	**Preauthorization & access**; insurer preauth causes delays/denials; Medicare protective vs. other payers.
Broglio et al. 2017	North America (USA-national)	**Military & Defense Health Systems** (DoD); **Multilateral Coalitions/Advocacy Platforms** (NCAA)	**Concussion research network**; NCAA-DoD CARE Consortium design & governance; >25k athletes enrolled across sites.
Kuo et al. 2016*	East Asia (Taiwan)	**National Ministries & Public Health Agencies** (traffic/health authorities; trauma registries)	**Helmet policy & outcomes**; helmet use linked to lower mortality/ICU need/complications (adjusted ORs); supports enforcement & prevention.
Sharma et al. 2017	North America (Canada-Ontario)	**National Ministries & Public Health Agencies** (MOHLTC; provincial program)	**System redesign/PNO**; centralized neurosurgery services with teleradiology/outreach; bundled funding & transfer coordination.
Russell et al. 2017	Africa (continental overview)	**Multilateral Coalitions/Advocacy Platforms** (IBRO, WFNS, WFN, ILAE)	**Neuroscience history/capacity**; charts growth of African neuroscience; highlights roles of IBRO/WFNS/WFN/ILAE and need for sustained training/funding links.
Menger et al. 2015	North America (USA-national)	**Health Financing & Reimbursement Bodies** (CMS); **National Ministries & Public Health Agencies** (policy context)	**ACA & value agenda**; argues for specialty registries (N2QOD) to define quality/value; urges unified, data-driven advocacy.
Middleton et al. 2015	Oceania (Australia & New Zealand)	**Multilateral Coalitions/Advocacy Platforms** (ANZ networks; research consortia); **National Ministries & Public Health Agencies** (government funders/policy)*	**SCI research strategy**; proposes networks/partnerships, priority-setting & consumer engagement; national registries/infrastructure roadmap.
Kondo et al. 2015	East Asia (Japan)	**National Ministries & Public Health Agencies** *(MHLW/public health programs)*	**Folic acid & NTDs**; FA awareness increased supplement use; recommends mandatory food fortification to lower spina bifida risk
Strom et al. 2013	North America *(USA — Louisiana)*	**National Ministries & Public Health Agencies** *(State helmet law; NHTSA; Louisiana Highway Safety Commission)*	**Motorcycle TBI prevention** - repeal raised fatalities; reinstatement increase helmet use but fatalities stayed high; recommends added policy/enforcement.
Groman et al. 2013	North America *(USA)*	**Health Financing & Reimbursement Bodies** *(CMS; CMMI; IPAB; PCORI)*	**Payment reform/quality** - ACA shifts reimbursement toward value; VBP, readmission/HAC penalties, physician value-modifier; need better specialty-relevant metrics and data infrastructure.
Cheng et al. 2011	East Asia (Taiwan)	**Health Financing & Reimbursement Bodies** (National Insurance Schemes — Taiwan NHI)	**Data governance/claims registries** - describes standardized NHI datasets and linkage for research & policy; notes application/review processes and privacy controls.
Khalessi et al. 2010	North America (USA)	**National Ministries & Public Health Agencies** (AHRQ/DHHS; CMS)	**Stroke (mechanical thrombectomy)/reimbursement** - formal response to AHRQ Draft Technical Brief; advocates evidence-based Medicare coverage; coordinates multispecialty input
Emejulu 2008	West Africa (Nigeria	**National Ministries & Public Health Agencies** (MOH; public tertiary hospitals)	**Workforce/capacity** - ~15 practicing neurosurgeons/150 M; services rated inadequate; calls for training expansion, facilities, enabling policy
Bean 2005	North America & East Asia (USA, Japan - OECD compari son)	**National Ministries & Public Health Agencies** (national health systems; OECD context)	**Health-system comparison** - Japan’s universal insurance + price controls vs. U.S. market pricing; argues neurosurgery must show cost-effectiveness to inform coverage
Holst et Cassidy 2004	Global	**Global Health Agencies** *(WHO Collaborating Centre Task Force on MTBI)*	**MTBI policy framework** - establishes task force; best-evidence synthesis on epidemiology/diagnosis/prognosis/treatment/costs; guidance to reduce burden.
Borg et al. 2004	Global	**Global Health Agencies** (WHO Collaborating Centre Task Force)	**MTBI care & costs** - early educational info and activation help; intensive routine follow-up not supported; indirect costs exceed direct.
Bean 2002	North America (USA)	**Health Financing & Reimbursement Bodies** (CMS/HCFA; AMA CPT; RUC)	**Valuation mechanics** - Explains CPT-RUC-CMS linkage; Medicare fee schedule becomes de-facto national schedule shaping neurosurgery payments
Khamlichi 2001	Africa (continental overview; North, West, East, Southern Africa)	**Global Health Agencies** (WHO African Subcommittee/WFNS link)	**Workforce & training** − 565 neurosurgeons across Africa; major equipment gaps; priorities: local training, instruments, CT access, shunts.
Antioch 2000	Oceania (Australia - Victoria)	**Health Financing & Reimbursement Bodies** (State health funding authorities)	**Hospital financing/casemix** - evaluates DRG/casemix purchasing and performance frameworks; recommends design goals and bundled/episode-based approaches for surgical services.
National Commission on the Protection of Human Subjects, 1977	North America *(USA - federal)*	**National Ministries & Public Health Agencies** *(DHEW; National Commission)*	**Psychosurgery policy** - proposed 2-yr moratorium debated; federal review instead; IRB oversight; informed consent; safeguards for vulnerable groups; national reporting

To contextualize policy evolution over time, Table 2 presents a timeline of major global milestones in neurosurgery governance, from the 2004 World Report on Road Traffic Injury Prevention to the 2025 Boston Declaration, highlighting the progressive formalization of neurosurgery within international health agendas.

**Table 2:** Timeline of major milestones in global neurosurgery policy

Year	Milestone	Description
2015	WHA 68.15	First global surgery resolution recognizing emergency and essential surgical care and anesthesia as part of universal health coverage.
2016	Bogotá Declaration	Launch of the Global Neurosurgery Action Plan and formal recognition of neurosurgery in global health dialogs.
2019	WHA 72 Follow-Up	Member states reviewed progress on implementation of WHA 68.15, with references to surgical workforce and governance.
2022	WHO IGAP	Intersectoral Global Action Plan on Epilepsy and Neurological Disorders (2022–2031), indirectly reinforcing neurosurgical priorities.
2025	Boston Declaration	Global pledges from over 100 GOs, NGOs, and academic institutions to expand neurosurgery collaboration and capacity.

### Global and regional governments

This group includes key health-related governmental organizations, such as the World Health Organization (WHO), its regional divisions, and the World Bank Group (WB). These organizations are fundamental in designing policy and supervising the delivery of neurosurgical care worldwide. The WHO is mentioned in about 25% of the reviewed literature, which implies its critical role in formulating policy direction on health that has vast effects on neurosurgery. The World Health Assembly (WHA), the governing body of WHO, sets its priorities, agenda, and budget. The WHO then engages with Member States’ Ministries of Health through WHO HQ, regional and national offices to facilitate better policies, health systems, and programs through an integrative approach to planning at the country level.

### National ministries of health

These agencies were mentioned in 23% of the articles as having an essential role in developing healthcare strategies appropriate for different countries concerning the needs for neurosurgery. They are instrumental in establishing the national system and setting healthcare trends that directly affect the provision of neurosurgical services. While East Africa and countries like India have started national neurosurgery programs to improve the quality of education and training of neurosurgeons, there are still many challenges in providing these services [9, 10]. 

### Insurance coverage and military service

Even though insurance services such as the Centers for Medicare and Medicaid Services (CMS) do not fund projects that deliver neurosurgical services, they influence how neurosurgical procedures are regulated and reimbursed, which has a significant effect on patient access and healthcare delivery in the US. They have been mentioned in 18% of our review search. 7% of our manuscripts mentioned U.S. military services and the importance of maintaining their implementation of neurosurgical procedures during warfare.

### International collaborative initiatives

This group encompasses transnational collaborations, including G4 Alliance, WHO Intersectoral Global Action Plan on Epilepsy and Other Neurological Disorders (IGAP), and Global Neurosurgery Initiative (GNI) of the Program in Global Surgery and Social Change (PGSSC) at Harvard Medical School, with direct neurosurgical involvement, and is mentioned in 5% of articles. In collaboration with GNI and G4 Alliance, Ecuador has been the first country in Latin America to launch a National Surgical Strengthening Plan, including UHC [11], paving the way for an equitable surgical care for the nation, but also putting pressure on the region to do the same [12]. 

## Discussion

### Global policy influence and governance mechanisms

The role of governmental organizations is key, not only in the development of policies, but also in their implementation across varying geographic regions. The expansion of the field in the past few decades point to the increased involvement of these organizations in advocating for the availability of neurosurgical services to all. This attitude is changing as Global Neurosurgery has become a more prominent discussion area [13]. Policy development and governance have played an essential role in the development of Global Neurosurgery. One of the most significant achievements was the WHA Resolution on Surgery and Anesthesia (2015) as UHC components [14]. Incorporating surgery into UHC is an important milestone in appreciating the need for surgical services within health systems and strengthening their capacity. In LMICs, insufficient health care coverage leads to staggering out-of-pocket expenses and reduces access to care. Therefore, the Lancet commission on Global Surgery 2015 underlined the importance of Universal Healthcare Coverage (UHC) for surgical professions such as Global Neurosurgery, thus making neurosurgery essential surgical care. They have estimated that a decrease in life-long disabilities and preventable deaths by introducing UHC is not only a sign of improved healthcare access, but is also cost-effective [15]. 

### Indirect public health pathways elevating neurosurgery

Beyond direct policy inclusion, neurosurgery has also gained legitimacy through indirect public health frameworks. Governmental influence on neurosurgical recognition has also occurred indirectly through broader public health agendas. In 2004, the WHO and WB, launched the “World Report on Road Traffic Injury Prevention,” which highlighted road traffic injuries as a worldwide public health priority, emphasizing that they were largely preventable. Traumatic brain injury treatment during warfare resembles LMICs’ resource limitations. It therefore ties directly into the global neurosurgical efforts to design global guidelines, data sharing, and equitable partnerships as the ENIGMA Brain Injury Workgroup suggests [16]. 

In that same year a United Nations General assembly passed resolution 58/289 (UN Road Safety Collaboration) to improve road safety [17]. In 2020, resolution A/RES/74/299 was made with the goal to decrease road traffic accidents by 50% by 2030, focusing on prevention and post-traumatic care [18]. The Global Status Report on Road Safety by the WHO in 2023 has shown that road traffic injuries are a leading cause of Traumatic brain injury and neurotrauma worldwide, particularly in LMICs, thus proving that Neurosurgery is essential surgical care and trauma systems need to be enhanced [19]. Over the years the road safety initiatives of the WHO have shown improvement in various nations such as 79% reduction of road traffic fatality in Tanzania and implementation of stricter helmet and seatbelt laws with reduction of fatalities in Brazil, Ethiopia, Vietnam etc [20]. For example, WHO and UN road safety frameworks between 2004 and 2023 increasingly framed traumatic brain injury as a preventable burden, indirectly positioning neurosurgical care as an essential component of emergency response systems.

### Capacity-building and NGO collaboration

While intergovernmental policies provide global direction, their implementation has relied heavily on hybrid NGO–government collaborations. Integrating neurosurgeons’ efforts for advocacy through research on existing global neurosurgical inequities has driven global politics in the healthcare domain. The WHO has launched several projects over the years to address some of these issues at a global scale, such as the *WHO Global Initiative for Emergency and Essential Surgical Care in 2005 (GIEESC)* [21] aims to reduce preventable death and disability from surgical conditions by improving emergency and essential surgical care in resource-limited settings. Although less frequently highlighted, these groups are doing critical work by advocating for neurosurgical access in LMICs and are steadily strengthening their position due to their advocacy and policy engagements. They focus on mobilizing resources and building capacity to close the gap in essential neurosurgical services [10, 22–24]. 

An example of this is found in Colombia’s development of a resource-stratified traumatic brain injury (TBI) management protocol (Beyond One Option for Treatment of Traumatic Brain Injury: A Stratified Protocol- The BOOTStraP) [25]. This protocol provides practical, resource-stratified algorithms for TBI care at all stages: prehospital, emergency department, surgery, and intensive care. The *IGAP* [26] in 2022, which focuses on transforming global care for epilepsy and neurological disorders by setting clear objectives and targets for countries to achieve over the next decade, with a strong focus on equity, multisectoral action, and measurable progress. The *76th WHA resolution on food micronutrient fortification* [27] in 2023, which solidified a global commitment to accelerate and expand food micronutrient fortification aims to prevent congenital disabilities, such as spina bifida, and other health consequences of vitamin and mineral deficiencies through evidence-based, large-scale interventions The resolution also supports the development of *National Surgical*,* Obstetric*,* and Anesthesia Plans (NSOAPs)* [28] for countries by developing national blueprints for expanding and improving surgical, obstetric, and anesthesia care, aiming to reduce preventable death and disability by integrating these services into national health systems and policies.

The developments would not have been possible without such collaboration, which has bettered the clinical setup in regions where the burden of disease for neurosurgical services is high but the provision of care is poor [29]. On the NGO side, organizations like the Global Neurosurgery Committee (GNC) of the World Federation of Neurosurgical Societies (WFNS), The Lancet Commission on Global Surgery, GNI of the PGSSC at Harvard Medical School, Global Alliance for Prevention of Spina Bifida (GAPSBiF), G4Alliance etc. have been of importance in launching these efforts. Still, significant challenges remain in improving data collection systems [2, 6, 15, 30].

### Persistent barriers to implementation

Despite the encouraging developments, there are still considerable challenges to advancing Global Neurosurgery. The diverse barriers to implementing these policies are particularly apparent in Sub-Saharan Africa, where there is a lack concrete national policies or strategies for neurosurgery-oriented services, due to lack of governmental funding for healthcare, training, infrastructure and resource resulting in high out-of-pocket expenditure for patients, lack of neurosurgical but also affiliated specialty workforce such as nurses, anesthesiologists, neurologists, intensive care units, bioengineering etc [31]. One of the most persistent barriers is the limitation of funding. Although the WHO’s Emergency and Essential Surgical Care Program has successfully advocated for neurosurgery, most countries still lack the resources to build neurosurgical units or train enough specialists [13, 32]. Sub-Saharan Africa remains a critical region where neurosurgery is severely under-resourced, with fewer than one neurosurgeon per 1.8 million people, far below the WHO’s recommended threshold of one per 100,000 [15, 33]. 

Another significant issue is the unavailability of dependable data, essential for effective policymaking and resource allocation. Many LMICs still lack the infrastructure to capture epidemiological data on TBI, cerebrovascular accidents, and other neurosurgical conditions. Without reliable data, governments and international organizations face difficulties formulating evidence-based policies, becoming aware of the economic impact of lacking specific policies, such as TBI, etc., and allocating resources where they are most needed [34–36]. The NGO, Mission: Brain and their Global Neurosurgery Research Group (GNRG) have fostered collaboration with the Sierra Leone Ministry of Health to bridge the gap with these services. Significant challenges remain in improving data collection systems, including technological and economic challenges [2, 6, 15, 30]. Infrastructure difficulties, such as lack of reliable water and electricity, transportation, weak healthcare systems, political instability, and allocation of resources, also limit timely access to neurosurgical care [13]. 

### Future directions and evolving global leadership

Despite these constraints, several developments indicate emerging resilience and renewed global momentum. Looking ahead, the Boston Declaration (April 2025) marks a significant step forward in fostering global leadership in neurosurgery and integrating it fully into the health sector. Governments will continue to play a central role in advancing the field of Global Neurosurgery and prioritization of collaboration of NGOs and academic institutions with national health systems.

There is still a need for increased training opportunities, improved healthcare infrastructure in LMICs, and innovative capacity building strategies to ensure that neurosurgical services remain sustainable in these regions. The role of international partnerships will remain crucial. The success of partnerships and the collaboration between government agencies provides a strong model for future cooperation between governments and global agencies. Moreover, strategic investments in data systems will allow governments and international agencies to track and assess the impact of neurosurgical interventions more effectively. Sadly, the recent development of US withdrawal from the WHO in January 2026 will affect funds to support WHO projects, as the US contributes 14% to the WHO’s budget [37]. The US has cut USAID funding, research funding, and support of institutions such as the National Institute of Health (NIH) and the Centers for Disease Control and Prevention (CDC). These cuts have significant effect on the reduction of other UN agencies budgets such as the United Nations Children’s Fund (UNICEF), United Nations High Commissioner for Refugees (UNHCR), United Nations Population Fund (UNFPA), World Food Program (WFP), etc., resulting in a budget reduction between 20 and 60% [38–41]. The US also withdrew from supporting Sustainable Development Goals (SDGs), including SDG3 on health. The US withdrawal from the WHO will most likely lead to an increase in nations’ healthcare system disruptions, reduction in training of specialists, resource development, and research in already marginalized regions, further increasing healthcare disparities. This hurdle emphasizes the necessity of collaboration with NGOs and academic institutions in LMICs to maintain the growth of Global Neurosurgery.

## Conclusion

In conclusion, the role of governments and international organizations in Global Neurosurgery cannot be overstated. These stakeholders are instrumental in developing policies, securing funding, and promoting partnerships that make neurosurgical care available to underserved populations. However, significant challenges remain, particularly regarding funding, data collection, and the persistent shortage of neurosurgical services in LMICs. The Boston Declaration (2025) provides a significant opportunity to highlight and advance the Global Neurosurgery agenda by strengthening policies and forging new international partnerships. In the coming years, governments and international organizations will need to pursue advancement of neurosurgical education, increased neurosurgical capacity and infrastructure, and creation of data management systems. Only through these concerted efforts will Global Neurosurgery be able to raise the needs of underserved populations in LMICs and ensure more equitable access to neurosurgical care.

## Supplementary information

Below is the link to the electronic supplementary material.

ESM 1 (DOCX 14.8 KB)

## Data Availability

No datasets were generated or analysed during the current study.

## Abbreviations

GNI: Global Neurosurgery Initiative
IGAP: International Global Action Plan on Epilepsy
WHA: World Health Assembly
TBI: Traumatic Brain Injury
WHO: World Health Organization
CMS: Centers for Medicare & Medicaid Services
NIH: National Institutes of Health
NS: Not Specified
NINDS: National Institute of Neurological Disorders and Stroke
