# Correction to: Governance and policy in global neurosurgery: a scoping review of national and international efforts

**DOI:** 10.1007/s10143-025-03997-x

**Published:** 2025-12-11

**Authors:** Ellianne dos Santos Rubio, Khalil St. Brice, Felix Toussaint, Jimena Colado-Martinez, Garrett W. Thrash, Ali Azan Ahmed, James Kelbert, Walt Johnson

**Affiliations:** 1 Department of Neurosurgery, Curaçao Medical Center, Willemstad, Curaçao Curaçao; 2 University of the West Indies, St. Augustine, Trinidad and Tobago; 3 American University of the Caribbean School of Medicine, Cupecoy, Sint Maarten; 4 Epilepsy Clinic, Institute of Neurology and Neurosurgery, México City, México; 5 Department of Neurosurgery, College of Medicine, University of Arizona, Tucson, AZ USA; 6 Program of Global Surgery and Social Change, Harvard Medical School, Boston, MA USA; 7 Department of Neurosurgery, Loma Linda University, Loma Linda, CA USA; 8 Mercy Ships, Garden Valley, Lindale, TX USA; 9 University of Alabama at Birmingham Heersink School of Medicine, Birmingham, AL USA; 10 Medical College, Aga Khan University, Karachi, Pakistan; 11 Global Neurosurgery Research Group, Mission:Brain Foundation, Sausalito, CA USA

**Correction to: Neurosurgical Review**

10.1007/s10143-025-03914-2

The authors regret that the name of **Ellianne dos Santos Rubio** was incorrectly captured as **Ellianne Santos dos Rubio** in the original published article. It is now corrected in this erratum article.

The original article online has been corrected.

