# Supplementary material for: Governance and policy in global neurosurgery: a scoping review of national and international efforts
Source: Neurosurg Rev. 2025 Oct 31;48(1):750. doi: 10.1007/s10143-025-03914-2 (PMC12578682; doi:10.1007/s10143-025-03914-2)
Supplement: Supplementary file 1 — (DOCX 14.8 KB) [file 10143_2025_3914_MOESM1_ESM.docx]

| **Database** | **Search String** | **Date Range** | **Results Retrieved** |
| --- | --- | --- | --- |
| PubMed | ((Government[MeSH Terms]) OR (Government Agency[MeSH Terms]) OR (Government Agencies[MeSH Terms]) OR (Government Programs[MeSH Terms]) OR (Government Regulation[MeSH Terms]) OR (Federal Government[MeSH Terms]) OR (Governmental Officials[MeSH Terms]) OR (Government Publications[MeSH Terms]) OR (Government Financing[MeSH Terms])) AND ((Neurosurgery[MeSH Terms]) OR (Neurosurgical Procedures[MeSH Terms])) AND ((Public Health[MeSH Terms]) OR (Public Health Administration[MeSH Terms]) OR (Access to Health Care[MeSH Terms]) OR (Health Systems Agencies[MeSH Terms]) OR (Health Systems Plan[MeSH Terms]) OR (Community Health Systems[MeSH Terms]) OR (policy[Title/Abstract])) | Inception - Oct 2024 | 248 |
| Embase | \|  \| \| --- \|   (exp government/ OR exp government agency/ OR exp federal government/ OR exp government regulation/ OR exp government publication/ OR exp government finance/ OR exp public health administration/ OR exp national health program/) AND (exp neurosurgery/ OR exp neurosurgical procedure/) AND (exp public health/ OR exp health care access/ OR exp health care planning/ OR exp community health care/ OR policy.ti,ab.) | Inception - Oct 2024 | 35 |
| Global Index Medicus | (neurosurgery) AND (policy OR governance OR ministry OR government) | Inception - Oct 2024 | 70 |
| WHO IRIS & Grey Literature | “neurosurgery” combined with “government”, “policy”, and “ministry of health” (manual keyword search) | Inception - Oct 2024 | 50 |

Supplementary Table 1. Full Search Strategy Across Databases
